# Alpha-Synuclein in the Regulation of Brain Endothelial and Perivascular Cells: Gaps and Future Perspectives

**DOI:** 10.3389/fimmu.2021.611761

**Published:** 2021-02-19

**Authors:** Tizibt Ashine Bogale, Gaia Faustini, Francesca Longhena, Stefania Mitola, Marina Pizzi, Arianna Bellucci

**Affiliations:** ^1^Division of Pharmacology, Department of Molecular and Translational Medicine, University of Brescia, Brescia, Italy; ^2^Biotechnology Division, Department of Molecular and Translational Medicine, University of Brescia, Brescia, Italy; ^3^Laboratory for Preventive and Personalized Medicine, Department of Molecular and Translational Medicine, University of Brescia, Brescia, Italy

**Keywords:** Parkinson's disease, α-synuclein, endothelial cells, blood brain barrier, border-associated macrophages, perivascular cells

## Abstract

Misfolded proteins, inflammation, and vascular alterations are common pathological hallmarks of neurodegenerative diseases. Alpha-synuclein is a small synaptic protein that was identified as a major component of Lewy bodies and Lewy neurites in the brain of patients affected by Parkinson's disease (PD), Lewy body dementia (LBD), and other synucleinopathies. It is mainly involved in the regulation of synaptic vesicle trafficking but can also control mitochondrial/endoplasmic reticulum (ER) homeostasis, lysosome/phagosome function, and cytoskeleton organization. Recent evidence supports that the pathological forms of α-synuclein can also reduce the release of vasoactive and inflammatory mediators from endothelial cells (ECs) and modulates the expression of tight junction (TJ) proteins important for maintaining the blood–brain barrier (BBB). This hints that α-synuclein deposition can affect BBB integrity. Border associated macrophages (BAMs) are brain resident macrophages found in association with the vasculature (PVMs), meninges (MAMs), and choroid plexus (CPMs). Recent findings indicate that these cells play distinct roles in stroke and neurodegenerative disorders. Although many studies have addressed how α-synuclein may modulate microglia, its effect on BAMs has been scarcely investigated. This review aims at summarizing the main findings supporting how α-synuclein can affect ECs and/or BAMs function as well as their interplay and effect on other cells in the brain perivascular environment in physiological and pathological conditions. Gaps of knowledge and new perspectives on how this protein can contribute to neurodegeneration by inducing BBB homeostatic changes in different neurological conditions are highlighted.

## Introduction

Neurodegenerative diseases represent a relevant health burden, especially considering the growing population of elderly subjects. Cerebrovascular disorders such as stroke are considered among the major predisposing factors for the development of neurodegenerative diseases, including Alzheimer's disease (AD) and Parkinson's disease (PD) (1, 2). In particular, PD is the second most common neurodegenerative disorder, affecting 2–3% of the population over the age of 65 years (3). The lack of knowledge on the molecular underpinnings of PD still limits the development of efficient therapies.

Protein aggregates enriched in insoluble α-synuclein fibrils and loss of dopaminergic neurons in the nigrostriatal system are key pathological features of this disorder (4, 5). Of note, the pathological deposition of insoluble α-synuclein at synapses is believed to act as the *primum movens* for neuronal degeneration in PD, as by hindering neurotransmitter release, it can trigger synaptic failure (6–8). This event can then negatively impinge on axonal projections, thus slowly flowing in a retrograde neurodegenerative process culminating in neuronal cell death (6–8). Additionally, α-synuclein-related neuroinflammation, microglia activation, and vascular degeneration (9–12) have been described as important players in disease pathogenesis. This notwithstanding, whether α-synuclein communicates with other neurovascular components such as border-associated macrophages (BAMs) and vascular endothelial cells (ECs), which are involved in the early phases of ischemic brain damage (13–16), remains to be explored.

Alpha-synuclein is a 14 kDa protein owning an undefined structure in aqueous solutions (17). In neurons, the protein regulates various processes including synaptic function, mitochondrial homeostasis, autophagy/lysosomal functions, and cytoskeletal reorganization (8, 18–24). The diverse domains of α-synuclein and its conformational plasticity allow the interaction with a plethora of other proteins and lipid membranes (20). Alpha-synuclein can also undergo post-translational modifications as amino-terminal and carboxy-terminal nitration and phosphorylation [e.g., Ser129 phosphorylation; (25–27)], which in turn can impact its conformation and can lead to the formation of toxic oligomers and fibrils (20). While oligomers can affect membrane permeability as well as neuronal excitability and engulf protein degradation systems (28–30), fibrils can disrupt the integrity of intracellular organelles and induce chronic inflammation (28, 31). In the brain, α-synuclein is expressed not only in the neuronal cells, but at lower levels also in astrocytes, macrophages, and the microglia (32, 33). In the periphery, the protein is expressed in red blood cells (34, 35), platelets (36), and in other immune cells, such as T cells, B cells, natural killer (NK) cells, and monocytes (32). It has been found that α-synuclein can bind microglia cell surface receptors, thus activating intracellular pathways mediating the release of cytokines and upregulating of proinflammatory genes (10, 37). The protein can also regulate ECs function by blocking the exocytosis of Weibel-Palade bodies (WPBs) (38) and by downregulating the expression of tight junction (TJ) proteins (39).

The deposition of α-synuclein insoluble aggregates named Lewy bodies (LB) or glial cytoplasmic inclusions (GCI) characterizes the brain of patients affected by PD and dementia with LB (DLB) or multiple system atrophy (MSA), respectively (5, 40). For this reason, these disorders are commonly referred to as synucleinopathies. Certain pathological strains of α-synuclein, by moving between the brain cells and across the blood–brain barrier (BBB) interfaces and acting as imprinting templates for the pathological conformational shift of other α-synuclein molecules, are believed to mediate the propagation of pathological aggregates within the brain, from the periphery to the brain, or from the brain to the periphery, with a prion-like fashion (41, 42).

This review focuses on how α-synuclein impacts vascular ECs and BAMs regulation and crosstalk. Current gaps and future perspectives in the context of neurological disorders are also presented.

## Alpha-Synuclein Functions in the Central Nervous System (CNS)

To date, the physiological function of α-synuclein has not been fully disclosed, but we know that it controls neurotransmitter release and synaptic plasticity, particularly inhibiting dopamine overflow and modulating synaptic vesicles storage (20, 43, 44).

The full-length α-synuclein isoform consists of 140 amino acids and its structure can be divided into three main regions. The N-terminal part is essential for membrane binding (45–47) and includes the sites of main familial PD mutations, A30P, A53T, and E46K (18, 20), as well as for several post-translational modifications (48). The central domain, called non-amyloid component (NAC), is hydrophobic and highly aggregation-prone (49), and is necessary and sufficient for α-synuclein fibrillation (50). Finally, the C-terminal region is enriched in negative charges (51) and can interact with the N-terminal domain to form a compact aggregation-resistant structure (52).

Alpha-synuclein is described as an intrinsically disordered protein as it can be found in monomeric form (53) or in a stable tetramer (54) when purified at neutral pH. Rapid environmental changes can induce the formation of partially folded intermediates or kinetically trapped transition states (55). Along aging, the high plasticity of α-synuclein, coupled with post-translational modifications and protein enrichment at synaptic sites, can promote in concert the formation of high molecular weight soluble or insoluble aggregates, such as oligomers, protofibrils, or fibrils (20, 21). In PD, α-synuclein deposition is thought to play a pathogenic role in triggering both central and peripheral neurons degeneration, thus underlying the onset of motor and non-motor symptoms, respectively (56, 57). Interestingly, both monomeric and aggregated α-synuclein can be transferred from cell-to-cell (neuron-neuron, neuron-glia), and also across the BBB, thus contributing to neuropathology spreading (58). Endocytosis, carrier-mediated transports, and tunneling nanotubes are described as the main mechanisms for these exchanges (59, 60). In addition, impairment in glymphatic transport and lymphatic drainage pathways results in the accumulation of α-synuclein in the brain parenchyma and the progression of PD-like pathology in transgenic mouse models (61). This is in line with evidence supporting that general systemic circulation would act as a route for long-distance transmission of endogenous α-synuclein (62).

Alpha-synuclein can also exert a physiological regulatory action on intracellular organelles, including mitochondria (19), endoplasmic reticulum (ER), mitochondria-ER associated membranes (63), Golgi apparatus (64), and nuclei (65). Although the nuclear localization of α-synuclein was the first to be reported, its involvement in DNA repair mechanisms has been described quite recently (66). Recent findings, showing reduced nucleus to cytoplasmic transport in induced pluripotent stem cell (iPSC)-derived neurons from familial patients with PD bearing A53T mutation or multiplication of the α-synuclein gene locus SNCA (67), support that the protein may also play a role in maintaining nuclear membrane functions. Interestingly, reduced α-synuclein DNA binding associates with transcription deregulation through inhibition of cell cycle-related genes and the nuclear localization of α-synuclein is modulated by its phosphorylation at Serine 129 (68).

The interplay between mitochondria and α-synuclein during the progression of PD still constitutes an issue to be solved, as the exact contribution of mitochondrial deficits and α-synuclein aggregation to dopaminergic neurons degeneration has yet to be clearly elucidated (69, 70). Indeed, the aggregation of α-synuclein induces neural deficits, but it is also evident that mitochondrial dysfunctions are crucial events in the pathogenesis of PD (71, 72). Notably, the observation that α-synuclein is increased following a stroke, and that its induction is involved in the response to post-stroke brain damage, reinforces the idea that the protein can act as a pivotal regulator of neuronal resilience to injury (35, 73–75).

Numerous studies have shown that α-synuclein accumulation and aggregation can activate neuroinflammation (76–79), in agreement with the evidence showing increased levels of tumor necrosis factor alpha (TNFα), interleukin-1β (IL-1β), and IL-6 in the brains of patients with PD (80–82). In particular, reactive microglia have been found in PD brains (83, 84) and in transgenic mouse models of PD, and can be activated by α-synuclein pathological deposition (85, 86). The main mechanisms involved in α-synuclein aggregates-related microglia response are the activation of nod-like receptor (NLR) pyrin domain containing 3 (NLRP3) or caspase 1 inflammasome and nuclear factor-κB (NF-κB) signaling (87). Moreover, the nicotinamide adenine dinucleotide phosphate (NADPH) oxidase (NOX) pathway has been found to modulate the migration of microglial cells exposed to the protein (88, 89). Of note, lipopolysaccharide (LPS) and IL-1β increase the expression of α-synuclein in human macrophages (90, 91), while murine macrophages are activated by full length α-synuclein *in vitro* and *in vivo* (92, 93). Finally, the expression of α-synuclein in peripheral blood mononuclear cells (PBMCs) (94) and its modulation in PD brains support that α-synuclein may be implicated in the modulation of systemic inflammatory responses, even though its exact contribution is to be further investigated (32).

## Alpha-Synuclein in ECs

**Figure 1:**
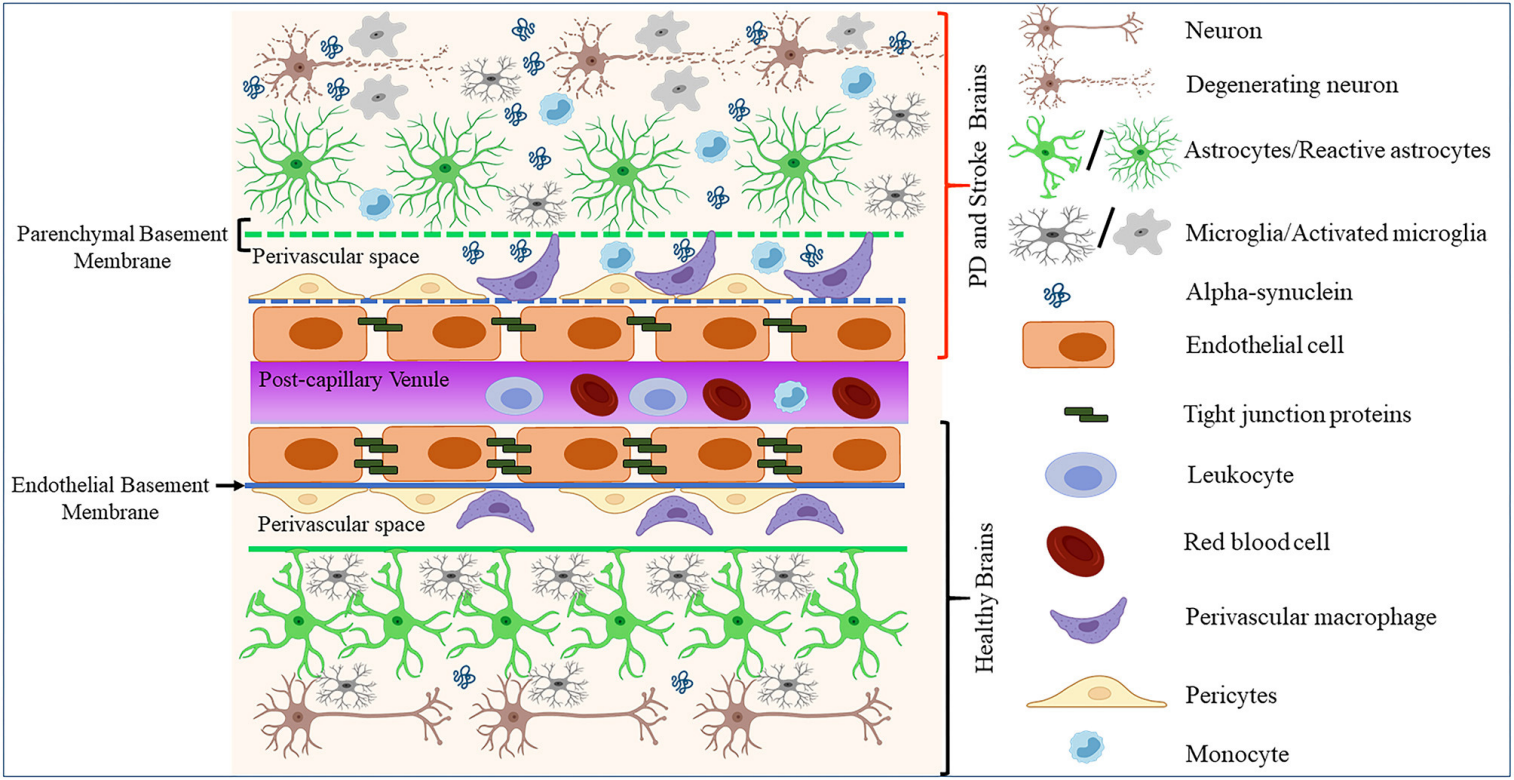
Schematic representation of how pathological α-synuclein-accumulation occurring in PD or stroke can disrupt the physiological homeostasis of the BBB by affecting its diverse components. The BBB is composed of microvascular endothelial cells, pericytes, astrocytes, and BM components deposited by ECs (endothelial BM) and astrocytes (parenchymal BM). More recently, perivascular macrophages and vessel-associated microglia were found to play a role in the maintenance and repair of BBB whose disruption is detected in various neurological disorders including stroke and PD. This could result in BM damage (dotted lines), downregulation of TJ proteins, abnormal accumulation, and spreading of toxic forms of proteins such as α-synuclein, activation of glial cells and PVMs, and infiltration of peripheral leukocytes and monocytes, leading to neuronal degeneration.

Endothelial cells constitute a distinct cell population coating the innermost lining of blood and lymphatic vessels (95, 96). These cells are known to exhibit differential gene expression, morphology, and function across the vascular tree and organs of the body (97, 98). However, to which extent such heterogeneity impacts the endothelial dysfunctions in neurodegenerative diseases such as PD remains unclear. Cerebral ECs exert multiple functions, including the formation of the BBB, the regulation of immune cells trafficking and vascular hemostasis, and the control of cell migration and proliferation (95). In the CNS, ECs organize and maintain the BBB through anatomical and molecular interactions with neurons, pericytes, astrocytes, microglia, and perivascular macrophages in the neurovascular unit (NVU) [(99–103); Figure 1]. Moreover, ECs and astrocytes secrete and deposit basement membranes (BM) that provide additional barrier functions [(104); Figure 1]. Interestingly, studies in stroke and PD models showed that BBB disruption leads to enhanced neuroinflammation and accumulation of toxic forms of α-synuclein, which in turn could promote the progression of neuronal loss by impacting on diverse components of the BBB [(73, 105); Figure 1].

The expression of α-synuclein in vascular ECs supplying the brain and peripheral organs has been known for a long time (96). In the normal human brain, a gradient distribution appears to exist, where α-synuclein is present in higher levels in ECs of leptomeningeal vessels, while intra-parenchymal and capillary ECs show lower and no expressions, respectively (106). Nonetheless, the existence of such graded expression in PD, its functional relevance, and regulation have not been elucidated yet. Conversely, ECs lines, including those derived from cerebral micro-vessels, exhibit low endogenous α-synuclein levels when compared to neurons (38, 39, 106).

**Figure 2:**
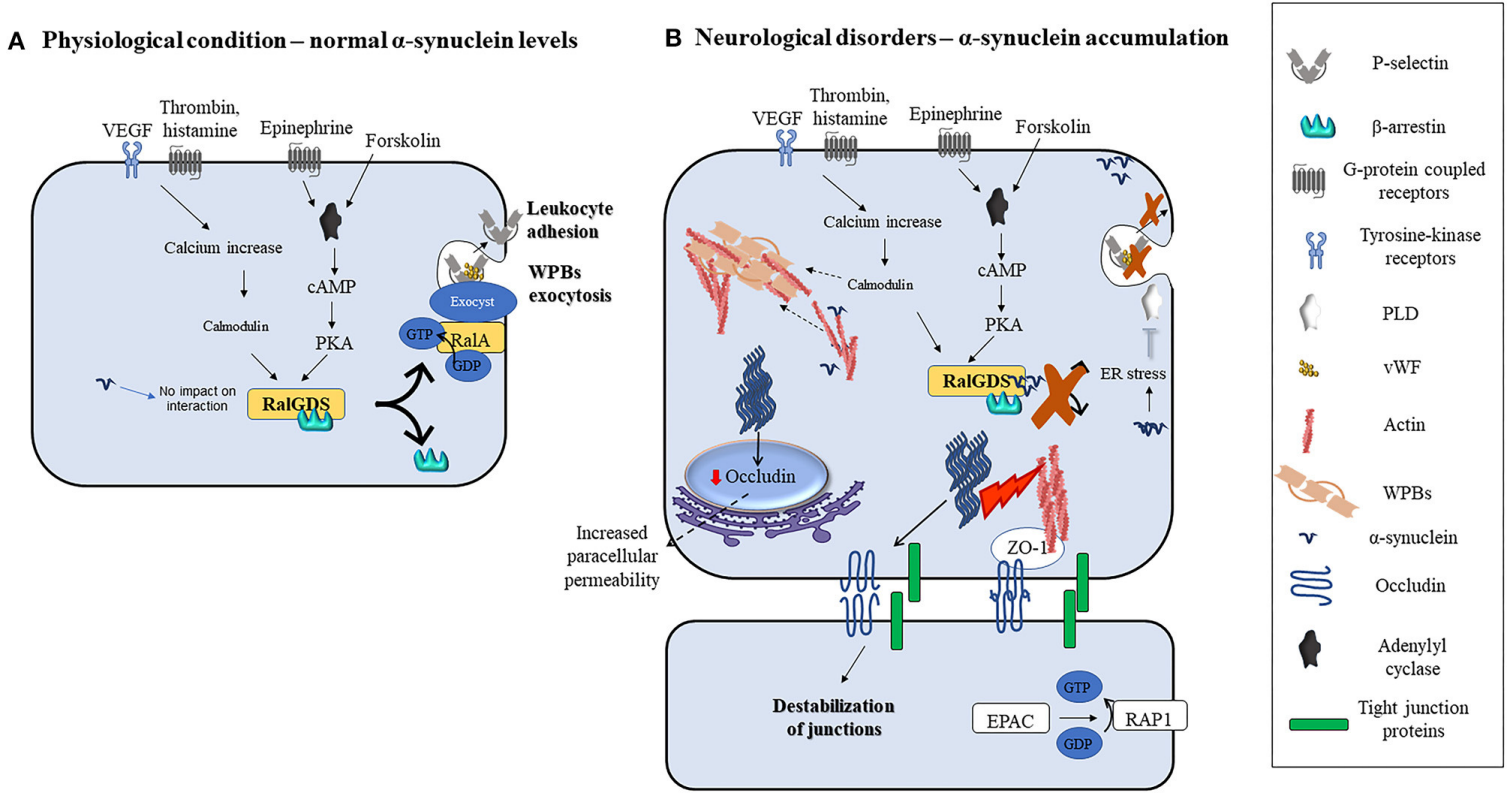
Modulation of ECs function by α-synuclein in physiological or pathological conditions. **(A)** Various agonists bind to endothelial cells G-protein coupled receptors (thrombin, superoxide, histamine, and epinephrine) or tyrosine kinase receptors (VEGF) to activate different intracellular pathways that lead to the release of contents of WPBs and translocation of P-selectin leading to leukocyte adhesion. Thrombin, VEGF, histamine, and superoxide lead to intracellular calcium increase and calmodulin activation, while forskolin and epinephrine increase intracellular cAMP and activate PKA. Calmodulin and PKA then trigger the translocation of RalGDS from cytosol to plasma membrane that activates membrane-bound RalA by exchanging GDP with GTP. This results in WPBs exocytosis. **(B)** Alpha-synuclein inhibits the release of contents of WPBs *in vitro* by various putative mechanisms which include binding to RalGDS and β-arrestin to prevent their dissociation, and hence exchange of RalA, blocking PLD activity, and immobilization of WPBs through enhanced actin polymerization. Fibrillary α-synuclein destabilizes TJ thereby affecting ECs paracellular permeability. This may occur through the downregulation of occludin and ZO-1.

Interestingly, transmitted-electron microscopy studies addressing subcellular localization in ECs, identified α-synuclein near WPBs, elongated intracellular granules that contain chemokines, cytokines, and adhesive molecules which are rapidly released into the extracellular space by agonists and modulate ECs response to stimuli (38). Pathological conditions such as hypoxia, ischemia, inflammation, and oxidative stress increase α-synuclein levels, its aggregation in neurons, and to some extent in non-neuronal cells *in vivo* and *in vitro* (35, 73, 74, 90, 107–109). However, similar stimuli failed to upregulate α-synuclein levels in ECs (106, 110), supporting the need for a better understanding of the mechanisms regulating its expression in these cells. Interestingly, wild-type and mutant α-synuclein inhibit the agonist-induced-release of von Willebrand factor (vWF) and P-selectin translocation from WPBs in ECs (38). These processes enable ECs to control vascular homeostasis during inflammatory response and thrombosis [(111, 112); Figure 2]. Indeed, agonists such as thrombin, vascular endothelial growth factors (VEGF), histamine, and superoxide can induce an increase in intracellular calcium levels. Subsequently, calcium binds and activates calmodulin which then triggers the translocation of Ral specific guanine exchange factor (RalGDS), from cytosol to plasma membrane and activates membrane-bound RalA (a small GTPase and substrate for RalGDS) by exchanging GDP with GTP [(112); Figure 2A]. Afterward, the RalA-GTP interacts with and assembles exocyst, a multi-protein complex important in targeting vesicles to membranes (113), to promote the exocytosis of WPBs (Figure 2A). In parallel, forskolin, or epinephrine can increase cyclic adenosine monophosphate (cAMP) levels thus inducing protein kinase A activation, which also causes RalGDS membrane translocation [(38, 112); Figure 2A]. Upon activation of these pathways, α-synuclein binds to both RalGDS and β-arrestin, thus enhancing their interaction and inhibiting their dissociation and translocation to the plasma membrane, thereby preventing WPBs exocytosis [(38); Figure 2B]. Moreover, it may be also feasible to foresee that exocytosis might also be prevented by inactivation of phospholipase D (PLD) due to α-synuclein overexpression-related ER stress (114) or enhanced polymerization of actin filaments by α-synuclein [(18, 115, 116); Figure 2B], that by immobilizing WPBs would avoid their release.

Alpha-synuclein is present in the cerebrospinal fluid (CSF) and the blood and transported across the BBB (59, 117–120). In particular, α-synuclein can be transferred by multiple transport mechanisms including carriers such as lipoprotein receptor-related protein-1 (LRP-1) (59) or extracellular vesicles (EVs) (121). Exosome-derived α-synuclein induces oligomerization of endogenous soluble protein in recipient cells and contributes to intercellular propagation of pathology. The CSF of patients with PD show α-synuclein containing exosomes derived from various cells including microglia and exert different functions (122, 123). In line with this, erythrocyte-derived exosomes containing α-synuclein from patients with PD induce microglial activation *in vivo* and *in vitro*, thus suggesting that erythrocyte-derived extravasated α-synuclein may play a role in disease pathogenesis (121). These evidences support that further studies are needed to understand how exosome-associated physiological or pathological forms of the protein may impact on brain immune cells and ECs function and thus on BBB integrity.

Alpha-synuclein-induced inflammation might contribute first to the stimulation of rapid ECs response, which by driving the contraction of ECs, leads to the formation of gaps between them. This reshaping of ECs alters the continuous ECs layer mediating the improvement of its paracellular permeability and induces the activation of ECs. Consequently, the induction of proinflammatory molecules production and release from ECs increases the local blood flow. These events, in conjunction with the ECs layer alteration, prompt BBB dysfunction, leading to the extravasation of protein-rich exudates as well as to the recruitment and activation of circulating leukocytes, that further promote neuroinflammation (124). In particular, it may be feasible that the chronic upregulation of TNF-α and IL-1β associated with α-synuclein deposition, observed in patients with PD and animal models (82), might induce sustained activation of ECs. The consequent activation of NF-κB and activator protein 1 (AP-1) and the production of vascular cell adhesion molecule 1 (VCAM 1) and intercellular adhesion molecule 1 (ICAM 1) (124), would thus set the stage for enhanced neuroinflammation, BBB injury, and neurodegeneration (Figure 2). On this line, the mechanisms linking α-synuclein deposition to endothelial injury warrants further investigation.

Evidence supports that α-synuclein preformed fibrils (*pffs*) downregulate the expression of occludin and of zonula occludens 1 (ZO-1) (Figure 2B). As a consequence, the transport across intercellular junctions between ECs could be improved (39). However, α-synuclein *pffs* do not trigger endothelial dysfunction or release of proinflammatory cytokines from ECs in culture (39), supporting that these cells are less vulnerable to α-synuclein toxicity. On the other hand, activation of Ras homologous guanosine triphosphate phosphatase (RhoGTPases) leads to distinct effects on the ECs' barrier function depending on the type of GTPase activated (125, 126). For instance, excess activation of RhoA by thrombin or VEGF induces the formation of stress fibers which destabilizes intercellular junctions and downregulates the expression of eNOS, thereby promoting paracellular permeability and endothelial dysfunction [(126); Figure 2]. A recent study on a human brain-chip modeling the *substantia nigra* (SN) showed that α-synuclein fibrils can induce increased paracellular permeability (127). Interestingly, transcriptomic analysis of the ECs in the brain-chip revealed the upregulation of genes involved in inflammation, oxidative stress, autophagy, efflux system, and extracellular matrix deposition and the downregulation of genes that encode for TJ proteins (127). Conversely, the overexpression of A30P mutated α-synuclein has been found to upregulate collagen IV α2 chain (COL4A2), a major constituent of BMs *in vivo* and *in vitro* (22), further supporting that α-synuclein changes may impact the BBB integrity also by affecting this component. However, whether and how α-synuclein influences these pathways to regulate ECs functions at the BBB or the secretion and assembly of other BM elements, their degrading enzymes, or interaction with receptor proteins and other neighboring cells still needs to be addressed. Likewise, since endothelial dysfunction might, in turn, alter the transport of α-synuclein between the brain and vasculature, thus promoting its accumulation and the progression of α-synuclein pathology, studies addressing whether and how BBB dysfunction may impact PD progression could bring new insights into our basic understanding of the pathophysiology of this disorder.

Indeed, brains of patients with PD show evidence of endothelial degeneration, downregulation of TJ proteins, and even angiogenesis (39, 105, 128, 129). These changes were observed mostly in the SN, *locus coeruleus* (LC), and *caudate putamen* (CP), brain regions where α-synuclein-induced degeneration is prominent, and to a lesser extent in the cerebral cortex (105, 128, 129). Moreover, pathological alterations in the capillary BM including collagen deposition and thickening are evident in PD brains (12, 128, 130). It is thus plausible to speculate that such changes may reduce the efficiency of the exchange of molecules between the brain and vasculature, rendering neurons vulnerable to oxidative stress and accumulating cellular waste products.

Angiogenesis is a well-recognized adaptive response to cerebral hypoxia or ischemia and is regulated by BM proteins and their integrin receptors (131). Interestingly, the integrin receptor αvβ is upregulated in angiogenic vessels (131, 132) and in cerebral vessels of patients with PD and incidental LB disease (iLBD) (129), suggesting that the immature nascent vessels generated in PD brains, could contribute to neuroinflammation by facilitating the infiltration of peripheral immune cells and inflammatory or toxic factors (129). Consistently, co-localization of areas of leakage of an intravascular tracer with β3 integrin-expressing new vessels, indicating the presence of both angiogenesis and compromised BBB, has been observed in toxin-induced animal model of PD (132). Based on Braak's PD staging (56), patients with iLBD may represent an early disease stage where LB is restricted to LC and SN (129). Therefore, the presence of angiogenesis in patients with iLBD and PD supports that α-synuclein related vascular dysfunction might precede or/and contribute to the progression of neuroinflammation and neurodegeneration. This is further substantiated by findings showing that α-synuclein-related angiogenesis and downregulation of TJ proteins are not necessarily related to inflammation (39, 129). Indeed, recent findings indicate that dysfunction in BBB accompanied by pathological activation of pericytes precedes the onset of neuronal degeneration in a mouse model of PD (133), thus supporting that vascular dysfunction may be an early pathogenic events leading to neuronal damage. Furthermore, VEGF plays a protective role in PD through a direct effect on dopaminergic neurons (134) or via the canonical VEGF receptor (VEGFR2) pathway (135). VEGF released by activated astrocytes and microglia acts in a paracrine fashion to modulate ECs structure and function both in PD and DLB patients and in animal models of PD (136). This notwithstanding, whether α-synuclein is involved in the upregulation of VEGF in ECs remains to be investigated.

**Figure 3:**
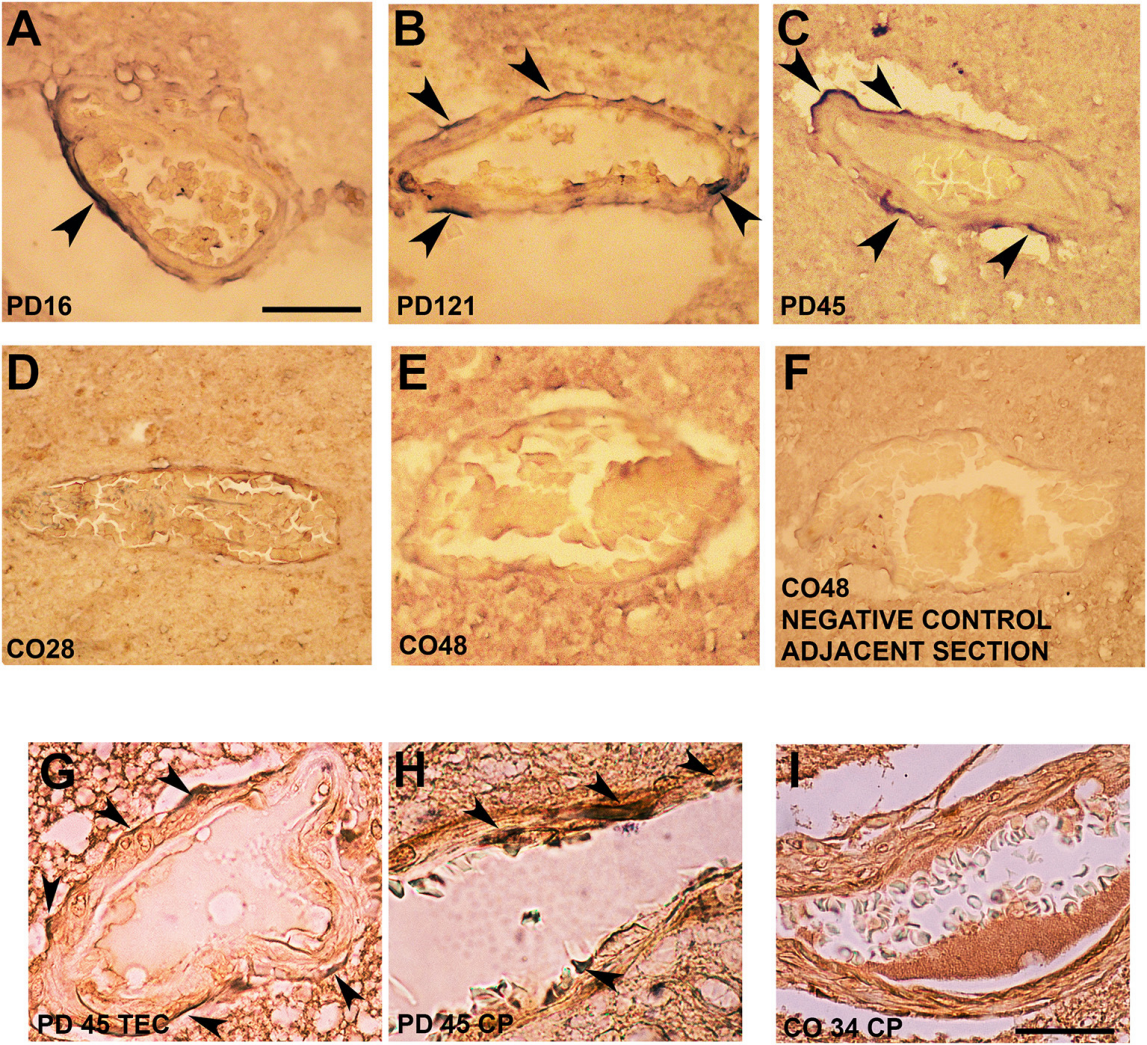
Alpha-synuclein perivascular immunoreactivity in *postmortem* sections from sporadic patients with PD. For these experiments, sections from three patients with PD (PD16, disease duration 18 years; PD45, disease duration 19 years; and PD121, disease duration 4 years) and three healthy controls (CO28, CO34, and CO48), kindly supplied by the Parkinson's UK Brain Bank, were analyzed. Briefly, sections were treated for antigen retrieval with 10 mM sodium citrate (20′ at 95°C) and 10% formic acid (15′ at RT). After 1 h incubation at room temperature (RT) with blocking solution (2% w/vol bovine serum albumin, 3% vol/vol normal goat serum, 0.3% Triton X-100 diluted in PBS 0.1 M pH 7.4) the 5-μm slices were subjected to either single α-synuclein (Sin211 MA5-12272 Thermo Fisher Scientific, Waltham, USA; dilution 1:500) or double laminin α2 (4H8-2, abcam ab11576; dilution 1:100)/α-synuclein (Sin211, MA5-12272 Thermo Fisher; dilution 1:500) immunolabeling according to previously described protocols (137, 138). Single α-synuclein immunopositive signal was revealed by Blue Alkaline Phosphatase (Vector Laboratories, Burlingame, CA) acquired by using a 40X objective, while for double immunolabeling laminin α2 was revealed by brown 3,3-diaminobenzidine (DAB) and α-synuclein by violet (Nickel supplemented) DAB (Vector laboratories) and acquired by a 100X objective. All the images were acquired by using an inverted light microscope (Olympus BX41; Olympus, Milan, Italy). **(A–C)** Representative images of perivascular α-synuclein immunolabeling (blue, arrows) in the TEC of three sporadic PD cases (PD 16, PD 121, and PD 45). **(D–F)** Images from the TEC of two of the healthy controls analyzed (CO28 and CO48). **(D,E)** The absence of α-synuclein immunolabeling in control brains, while **(F)** shows a representative image from a negative control for the immunostaining performed without the addition of the primary antibody on an adjacent section of CO48. **(G,H)** Representative images showing the presence of α-synuclein violet immunolabeling at the outer (**G**, arrows) and inner (**H**, arrows) side of laminin α2-positive perivascular BM in the brain of a sporadic patient with PD (PD45). Images are representative of the TEC **(G)** and CP **(H)**. **(I)** Representative image showing the absence of α-synuclein accumulation around laminin α2-immunolabeling in the proximity of a vessel of the CP of a healthy control (CO34). Scale bar: **(A–F)** 40 μm; **(G–I)** 25 μm.

Interestingly, in *postmortem* sections of the trans-entorhinal (TEC) cortex (Figures 3A–C) and CP (not shown) from sporadic patients with PD, we observed perivascular accumulation of α-synuclein immunoreactivity (in blue) in correspondence of some vessels. Conversely, the brains of healthy or negative controls did not exhibit this feature (Figures 3D–F). By double immunolabeling of laminin α2 (brown) and α-synuclein (violet), we found that in the PD brains, α-synuclein-positive perivascular staining could be identified either in the outer (Figure 3G) and inner (Figure 3H) sides of the perivascular basement membrane, while in control brains, it did not show α-synuclein positivity in the proximity of laminin α2 staining (Figure 3I).

**Figure 4:**
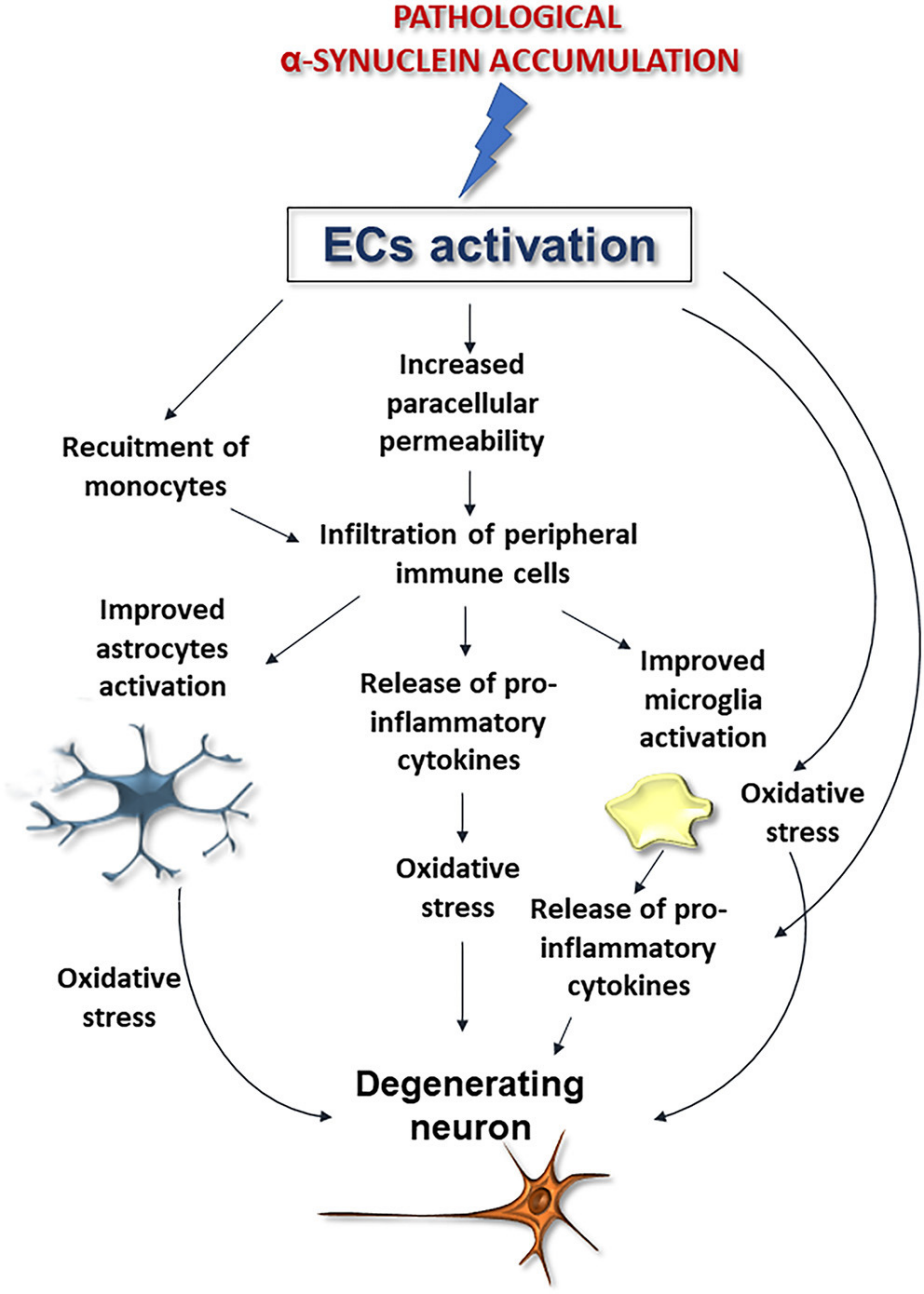
Possible downstream effects of pathological α-synuclein-induced activation of ECs on microglia, astrocytes, and neurons. Alpha-synuclein accumulation-induced ECs activation could promote BBB disruption and peripheral immune cell infiltration, improve microglia and astrocytes activation and exacerbate oxidative stress, thus promoting neuronal degeneration.

Although further studies are needed to corroborate whether and how α-synuclein deposition affects these cells, these findings, when coupled to the aforementioned noxious effects exerted by pathological α-synuclein on ECs, support that perivascular accumulation of the protein, by inducing ECs activation may compromise brain vessels integrity exacerbating astrocyte and microglia activation thus promoting neurodegeneration and BBB disruption (Figure 4).

## Alpha-Synuclein in BAMs and Other Perivascular Cells

Border-associated macrophages are a subset of CNS myeloid cells (macrophages) that like microglia originate prenatally in the yolk sac (139), invade the brain during the early prenatal period, and localize in the choroid plexus, perivascular, and leptomeningeal spaces. BAMs form stable populations with the sole exception of the choroid plexus macrophages that exchange with peripheral monocytes (139, 140). Indeed, BAMs can be anatomically distinguished into perivascular macrophages (PVM), meningeal macrophages (MAM), and choroid plexus associated macrophages (CPM) (139, 140). In healthy brains, PVMs are involved in the regulation of BBB permeability and phagocytosis of pathogens but can also promote the entrance of peripheral immune cells into the brain (102). Under pathological conditions such as cerebral amyloid angiopathy (CAA), AD (141) and PD (141), PVMs can participate in the clearance of toxic amyloid-β and α-synuclein. Consistently, the depletion of PVMs using clodronate-containing liposomes in a mouse model of PD resulted in the increased expression of VCAM 1, the infiltration of T cells, and the propagation of α-synuclein pathology (142). Moreover, in rats that underwent transient ischemia followed by reperfusion injury, BAMs were involved in promoting peripheral immune cell infiltration and vascular permeability without impacting the extent of ischemic damage, thus suggesting that additional studies are needed to fully understand the modulatory role of these cells in cerebrovascular dysfunctions and neuroinflammation (13).

Border associated macrophages are involved in immune surveillance and support the entrance of peripheral immune cells into the CNS under pathological conditions (143, 144). In rodents, monkeys, and humans, PVMs express the mannose receptor CD206 (145) and the scavenger receptor CD163, which under physiological conditions is expressed on tissue macrophages, with the exception of microglia and some monocytes (146–148). Recent research reports, dissecting the molecular signature of brain macrophages in mice at the single-cell level, reported a clear segregation of BAMs from microglia, identified a BAMs core gene signature, and even showed heterogeneity within BAMs (149–152). In stroke animal models and patients, a unique transcriptional signature of BAMs, their local proliferation and migration in the brain parenchyma, have been detected (14).

It is now believed that BAMs play a role in immune function, BBB integrity, and lymphatic clearance (139, 140). Currently, the identification of BAMs mainly relies on the use of anatomical studies aimed at disclosing their localization in the brain thanks to the use of few reliable molecular markers (139, 140). However, this approach is not applicable in the presence of inflammatory conditions or tissue injury when peripheral monocytes/macrophages enter the brain and reside in the same location and express similar molecular markers (139). Despite these limitations, remarkable progresses have been made to fully characterize and understand their role in the normal and diseased brain.

Alpha-synuclein is expressed by microglia and peripheral monocytes/macrophages in a lower amount compared to neurons (32), but the expression in BAMs has not been described yet. Since BAMs share similar ontogeny and molecular and immunologic characteristics with microglia (139, 140), they might exhibit analogous changes and activation states to α-synuclein stimulation (153, 154). Indeed, it has been described that BAMs and microglia display multiple similarities such as the expression of myeloid-specific markers. Among them, ionized calcium-binding adaptor molecule 1(Iba1), F4/80 (mouse) or EMR1 (human), chemokine receptors, scavenger receptors, receptor tyrosine kinases, Integrins, pattern recognition receptors (PRRs), and cytokines receptors (155). α-synuclein is known to induce inflammatory response and migration of microglia (32, 89, 156). For instance, previous studies showed that α-synuclein induces NOX2 activation in microglia by binding to toll-like receptor 2 (TLR-2) and CD11b leading to microglia-mediated neuronal toxicity (157). Similarly, α-synuclein binds to TLR-4 and activates NF-kB signaling which then induces a selective autophagy pathway named synucleinphagy and release of exosomes containing the protein, thus contributing to the intercellular spread of α-synuclein pathology (154).

Interestingly, monomeric α-synuclein can also impact microglia polarization by conferring an anti-inflammatory profile to these cells through the interaction with extracellular signal-regulated kinase (ERK) and the recruitment of the ERK/NF-κB, and peroxisome proliferator-activated receptor γ (PPARγ) pathways (158). It may thus be feasible that these pathways may also be activated upon exposure of BAMs to α-synuclein.

It is worth mentioning that pericytes can also play a role in the formation, maintenance, and regulation of BBB (159). Additionally, pericytes (60) and astrocytes (160) can mediate the transfer of α-synuclein between cells of the NVU, suggesting a possible role of non-neuronal cells in α-synuclein pathology spreading in PD (161). *In vitro* studies also showed that α-synuclein activates pericytes which in turn release proinflammatory mediators that can mediate BBB dysfunction (162). Early pericyte activation associated with BBB leakage has been recently described in a human α-synuclein overexpression-based mouse model of PD (133), thus further supporting that vascular pathology can constitute a relevant pathophysiological aspect of PD.

Peripheral immune cells such as lymphocytes have also been involved in the pathogenesis of PD. Indeed, studies in the brains of patients with PD and animal models showed that T cells with upregulated expression of the ICAM 1 receptor lymphocyte function-associated antigen-1 (LFA1) can promote leukocyte infiltration (163). Alpha-synuclein-specific T-cell reactivity has been found to be higher in early PD while decreasing in patients with late-stage disease (164). When considering the increase of α-synuclein in animal models of stroke (73), where Treg cells interact with ICAM1 on inflamed microvessels and platelets promoting vascular dysfunction (165), this evidence suggests that the accumulation of α-synuclein occurring following brain ischemia could very well-boost these pathogenic processes.

Several studies showed that α-synuclein aggregates can be detected in reactive astrocytes in the brains of patients with PD and animal models (33) suggesting a role of these cells in the clearance and /or spreading of α-synuclein toxicity in the brain parenchyma and NVU. Even though the role of endogenous α-synuclein in astrocytes remains to be fully explored, in disease states, α-synuclein activates astrocytes by interacting with PRRs such as TLR-4 (33). Activated astrocytes can in turn uptake and degrade the protein via the endosomal-lysosomal pathway and contribute to non-cell autonomous degeneration (166, 167).

Recent evidence suggests that α-synuclein can be removed from the brain via extracellular space drainage pathway which includes glymphatic transport and meningeal lymphatic system (61), whose reduction lead to the accumulation of toxic forms of amyloid-β in the brain parenchyma of AD rodent models (168–170). Similarly, a recent study in a transgenic PD mouse model overexpressing human A53T mutated α-synuclein showed that blockage of the deep cervical lymph node reduces glymphatic transport of an intraventricular tracer and promotes the accumulation of α-synuclein and its aggregation in SN, thus leading to the progression of α-synuclein pathology (61).

Taken together, impairment in these systems results in the accumulation of toxic proteins in the brain and contributes to the progression of neurodegenerative diseases. The close association of BAMs to perivascular and lymphatic drainage systems in the brain when coupled to the detection of α-synuclein aggregates in the perivascular space of a PD mouse model (61) supports that understanding of whether and how these cells contribute to the clearance of α-synuclein along these pathways deserves *ad-hoc* investigation.

In addition to this, it is plausible that the increase in α-synuclein levels observed following ischemia and spinal cord injury (35, 73, 74, 171) could result in a chemotactic gradient for microglia migration and activation (89) contributing to brain damage. Consistently, inhibition of α-synuclein induction following ischemia or spinal cord injury reduces secondary neuronal injury, inflammatory response, and improves neurological outcomes (171, 172). Although, it is known that juxta vascular microglia play a divergent role in repairing vascular injuries following an insult or systemic inflammation (173–175), whether α-synuclein modulates these cells or BAMs remains to be clarified.

## Alpha-Synuclein Role in Modulating BBB Cells Interaction

In the normal and diseased brain, ECs communicate with neurons, microglia, pericytes, and astrocytes to regulate vascular function (176). More importantly, the interaction of microglia with ECs can exert divergent roles in regulating BBB integrity (175). In co-cultures of ECs and neurons, α-synuclein fibrils resulted in endothelial dysfunction, but this effect was not observed in ECs monocultures (39), supporting that neuronal-ECs crosstalk at the NVU may be perturbed by pathological α-synuclein.

CD200, a transmembrane protein found to be expressed in neurons, astrocytes, oligodendrocytes, and ECs, transduces signal via its receptor (CD200R), expressed on myeloid cells including microglial and BAMs (177). CD200-CD200R and C-X3-C motif chemokine ligand 1 (CX3CL1)-C-X3-C motif chemokine receptor 1 (CX3CR1) signaling between neurons and microglia helps to maintain microglia in the resting state (178). Inactivation of the transmembrane glycoprotein CD200R in microglia of a toxin-induced PD mouse model results in increased activation of these cells, release of proinflammatory cytokines, loss of dopaminergic neurons in the SN, and behavioral deficits (179). Similarly, monocyte-derived macrophages (MDMs) from patients with PD show dysregulation in CD200R signaling (180).

On the other hand, M2 macrophages express pro-angiogenic factors such as VEGF and fibroblast growth factor 2 (FGF2), that by activating their receptors (VEGFR2 and FGFR) promote angiogenesis and neuronal survival (181). *In vitro* studies have shown that microglia maintains ECs in a resting state by secreting transforming growth factor-beta (TGF-β), an anti-inflammatory cytokine, while the proinflammatory TNF-α induces ECs proliferation (182). However, whether this kind of communication occurs also between ECs and BAMs or is influenced by α-synuclein still remains to be elucidated.

Secreted toxic species of α-synuclein are known to bind to various cell surface receptors in adjacent cells and activate several intracellular pathways leading to synaptic dysfunction, neurodegeneration, and inflammation (183). On this line, α-synuclein binds to TLR-2, TLR-4, and CD11β integrin to activate NF-kB signaling and assembly of NLRP3 inflammasome in microglia (37, 154, 157). It is thus plausible that various receptors for α-synuclein might exist in different cells, including ECs, and that the protein may regulate their intracellular activities or their crosstalk within neighboring cells such as BAMs.

## Current Gaps and Future Perspectives

While α-synuclein associated vascular dysfunction is evident in PD (11, 128), most of the studies aimed at understanding the physiological role of the protein in ECs have been performed by overexpressing the protein through transgene expression (38, 39). Therefore, the role of extracellularly released α-synuclein on these cells still needs to be extensively explored. Furthermore, it is not yet established whether ECs respond to various pathogenic stimuli by regulating α-synuclein level or its transport across the BBB. Future studies exploiting improved models might overcome these limitations.

It is now clear that α-synuclein-associated inflammation contributes to the pathophysiology of PD (10) but research on whether or how α-synuclein modulates the function of macrophages in the brain has been mostly focused on microglia. As a result, our knowledge on the role of α-synuclein in other brain resident macrophages such as BAMs or other perivascular cells is poor. Similarly, despite the presence of studies indicating interplay between ECs and microglia (175), the interplay between perivascular cells and ECs has been scarcely studied. For instance, since recent evidence showed that BAMs and microglia can acquire distinct genetic and molecular phenotypes early in development (152), studying whether α-synuclein plays a role in modulating the signaling pathways mediating the crosstalk between BAMs and ECs could bring novel and significant insights for understanding the biological basis of neurological disorders such as PD or stroke. Similarly, though α-synuclein transfer between cells and across BBB interfaces has been established (59, 154), whether and how BAMs are involved in this event also deserves further investigations.

A deeper understanding of the role of physiological and pathological forms of α-synuclein in the modulation of BAMs and ECs or their interplay may also greatly aid the identification of novel therapeutic targets for stroke or neurodegenerative synucleinopathies.

## Author Contributions

TB and GF wrote and revised the article and prepared the figures. TB performed immunostaining. FL revised the article and was involved in image acquisition. AB, MP, and SM did a critical revision of the article. All authors contributed to the article and approved the submitted version.

## Conflict of Interest

The authors declare that the research was conducted in the absence of any commercial or financial relationships that could be construed as a potential conflict of interest.

## Glossary

### Abbreviations

AD: Alzheimer's disease
AP-1: activator protein 1
BAMs: border associated macrophages
BBB: blood–brain barrier
BM: basement membrane
CAA: cerebral amyloid angiopathy
cAMP: cyclic adenosine monophosphate
CD200R: CD200 receptor
CNS: central nervous system
COL4A2: collagen IV alpha 2 chain
CP: *caudate putamen*
CPM: choroid plexus associated macrophages
CSF: cerebrospinal fluid
CX3CL1: C-X3-C motif chemokine ligand 1
CX3CR1: C-X3-C motif chemokine receptor 1
DAB: 3,3-diaminobenzidine
DLB: dementia with LB
ECs: endothelial cells
ER: endoplasmic reticulum
ERK: extracellular signal-regulated kinase
EVs: extracellular vesicles
FGF2: fibroblast growth factor 2
FGFR: FGF2 receptor
GCI: glial cytoplasmic inclusions
Iba1: ionized calcium-binding adaptor molecule 1
ICAM 1: intercellular adhesion molecule 1
iLBD: incidental LB disease
IL: interleukin
iPSC: induced pluripotent stem cell
LB: Lewy bodies
LC: *locus coeruleus*
LFA: lymphocyte function-associated antigen-1
LPS: lipopolysaccharide
LRP-1: lipoprotein receptor-related protein-1
MAM: meningeal macrophages
MDMs: monocyte-derived macrophages
MSA: multiple system atrophy
NAC: non-amyloid component
NADPH: nicotinamide adenine dinucleotide phosphate
NF-κB: nuclear factor-κB
NK: natural killer
NLR: nod-like receptor
NLRP3: NLR pyrin domain containing 3
NOX: NADPH oxidase
NVU: neurovascular unit
PBMCs: peripheral blood mononuclear cells
PBS: phosphate buffer saline
PD: Parkinson's disease
*pffs*: preformed fibrils
PLD: phospholipase D
PPARγ: peroxisome proliferator-activated receptor γ
PRRs: pattern recognition receptors
PVM: perivascular macrophages
RalGDS: Ral specific guanine exchange factor
RhoGTPases: Ras homologous guanosine triphosphate phosphatase
RT: room temperature
SN: *substantia nigra;*
TEC: trans-entorhinal cortex
TGF-β: transforming growth factor-beta
TJ: tight junction
TLR-2: toll-like receptor 2
TLR-4: toll-like receptor 4
TNFα: tumor necrosis factor alpha
VCAM 1: vascular cell adhesion molecule 1
VEGF: vascular endothelial growth factors
VEGFR2: VEGF receptor
vWF: Willebrand factor
WPBs: Weibel-Palade bodies
ZO-1: zonula occludens 1.
